# Targeting MYC activity in double-hit lymphoma with *MYC* and *BCL2* and/or *BCL6* rearrangements with epigenetic bromodomain inhibitors

**DOI:** 10.1186/s13045-019-0761-2

**Published:** 2019-07-09

**Authors:** Weiping Li, Shiv K. Gupta, Weiguo Han, Ryan A. Kundson, Sara Nelson, Darlene Knutson, Patricia T. Greipp, Sherine F. Elsawa, Eduardo M. Sotomayor, Mamta Gupta

**Affiliations:** 1 Department of Hematology, Mayo Clinic, Rochester, MN USA; 2 Department of Radiation Oncology, Mayo Clinic, Rochester, MN USA; 3 Department of Laboratory Medicine and Pathology, Mayo Clinic, Rochester, MN USA; 4 Department of Molecular, Cellular and Biomedical Sciences, University of New Hampshire, Durham, NH USA; 5 Department of Biochemistry and Molecular Medicine, School of Medicine and Health Sciences, George Washington University, GW Cancer Center, Washington, DC 20052 USA

**Keywords:** DLBCL, Double-hit lymphoma, MYC, BCL2, BET bromodomain, BRD4

## Abstract

Double/triple-hit lymphomas (DHL/THL) account for 5–10% of diffuse large B cell lymphoma (DLBCL) with rearrangement of *MYC* and *BCL2* and/or *BCL6* resulting in MYC overexpression. Despite the poor prognosis of DHL, R-CHOP chemotherapy remains the treatment backbone and new targeted therapy is needed. We performed comprehensive cytogenetic studies/fluorescence in situ hybridization on DLBCL and Burkitt lymphoma cell lines (*n* = 11) to identify the DHL/THL DLBCL in vitro model. We identified MYC/IG in Raji and Ramos (single hit); MYC/IG-BCL2 (DHL) in DOHH2, OCI-LY1, SUDHL2, and OCI-LY10; MYC/IG-BCL2/BCL6 (THL) in VAL; and no MYC rearrangement in U2932 and HBL1 (WT-MYC). Targeting MYC in the DHL/THL DLBCLs through bromodomain extra-terminal inhibitors (BETi) (JQ1, I-BET, and OTX015) significantly (*p* < 0.05) reduced proliferation, similar to WT-MYC cells, accompanied by decreased MYC but not BCL2 protein. Moreover, BETi suppressed MYC transcription and decreased BRD4 binding to MYC promoter in DHL cells. CD47 and PD-L1 are immunoregulatory molecules often expressed on tumors and regulated by *MYC*. High levels of surface CD47 but not surface PD-L1 was observed in DHL/THL, which was reduced by JQ1 treatment. BETi in combination with Pan-HDAC inhibitor had a limited effect on survival of DHL/THL, while combination of BETi and BCL2 inhibitor (ABT-199) had a significant (*p* < 0.005) inhibitory effect on survival followed by BCL-XL inhibition. Overall, the data suggests that MYC-expressing DLBCLs are probably addicted to the MYC-oncogenic effect regardless of MYC rearrangements. In summary, we identified an in vitro model for DHL/THL DLBCLs and provide evidence for the therapeutic potential of BET inhibitor alone or in combination with BCL2 inhibitor.

**Electronic supplementary material:**

The online version of this article (10.1186/s13045-019-0761-2) contains supplementary material, which is available to authorized users.

## Introduction

Diffuse large B cell lymphoma (DLBCL) is the most common aggressive B cell lymphoma in the USA. Based on gene expression profiling (GEP) studies, DLBCL can be classified into germinal center B cell (GCB) and activated B cell (ABC) subtypes [1]. In addition to the cell of origin, genetic studies have identified a prognostic role for *MYC* rearrangements in DLBCL. Earlier studies reported that 5–15% of DLBCL harbored *MYC*, *BLC2*, and/or *BCL6* translocations and were called “double-hit” lymphoma (DHL) or triple-hit lymphoma (THL). In the most recent WHO revision of lymphoma classification, DHL/THL category is now recognized as "high-grade B cell lymphoma (HGBL) with rearrangements of *MYC* and *BCL-2* and/or *BCL-6* [2]. In most DHL cases, *MYC* rearrangements (MYC/IGH or IGL, IGK) co-occur with *BCL-2* or *BCL-6*; however, in THL cases, *MYC* rearrangements (MYC/IGH or IGL, IGK) co-occur with both *BCL-2* and *BCL-6.* The DHL with *BCL-2* translocation has an aggressive clinical presentation and is hard to treat with conventional chemotherapy [3, 4]. The clinical behavior of DHL with *BCL-6* cases (*MYC/BCL6*) is not well understood. With standard therapeutic approaches such as with rituximab, cyclophosphamide, doxorubicin, and vincristine (R-CHOP) [5], DHL/THL groups have a prognosis worse than patients without MYC/IG rearrangements and the median overall survival for DHL/THL varied from 4.5 to 34 months [6–12]. There are some DLBCLs in which *MYC* and *BCL2* genes are overexpressed at the protein level, without genetic rearrangements. MYC protein expression is detected in a much higher proportion of DLBCL (around 40%) and is associated with concomitant expression of BCL-2 [13]. This profile was referred to as the “double-expresser” phenotype in the revised WHO classification of lymphoid neoplasms [2, 3, 14]. The double-expresser lymphomas have a worse outcome than other DLBCLs but they are not as aggressive as the HGBL, with rearrangements of *MYC* and *BCL-2* and/or *BCL-6* [3, 14].

Despite the poor prognosis in DHL, R-CHOP remains the backbone of treatment; it is an area of active preclinical and early-phase clinical research for exploring novel approaches for the treatment of difficult lymphomas. MYC and BCL2 translocations drive proliferation and prevent apoptosis in DHLs. We have previously shown that MYC overexpression correlated with inferior event-free survival in DLBCL [15]. MYC acts as a proto-oncogene and plays an important role in hematologic cancers such as aggressive B cell lymphoma [16] as well as in a number of solid tumors [17–21]. Despite the well-established role of MYC protein in driving cancer cell growth, no direct MYC-targeted therapeutic agent has advanced to the clinical setting for DHL and THL DLBCLs. Progress is being made in the targeting of the regulation of MYC activity by BET inhibitors in the MYC-expressing murine lymphoma or DLBCL cell lines [22–24]. However, very few studies described the BET protein role specifically in DHL/THL model. Potent and selective small molecule inhibitors of BET bromodomain are being clinically evaluated to target MYC in several diseases [25]. Therefore, in this study, we sought to identify DHL/THL cell lines and understand the role of BET bromodomain inhibition alone or in combination with other therapies in DHL/THL DLBCL.

## Materials and methods

### Human DLBCL cell lines

The B cell lymphoma cell lines OCILY10 (LY10), SUDHL2 (DHL2) OCILY1(LY1), OCILy3, and OCILy19 were a kind gift from Dr. Louis Staudt (NCI, Bethesda, MD, USA). VAL and U2932 cell lines were kindly provided by Dr. Izzidore Lossos (University of Miami, Miami, FL, USA). All cell lines were grown in Iscove’s modified Dulbecco’s medium supplemented with 20% human serum and antibiotics/antimycotics. Raji, Ramos (BL), and DOHH2 cell lines were purchased from ATCC (Manassas, VA) and were cultured in RPMI supplemented with 10% FBS.

### Antibodies and drugs

Antibodies to c-MYC, BCL-6, BCL-2, BCL-XL, MCL-1, P21, BIM, and H3K27Ac were obtained from Cell Signaling Technology (Beverly, MA). Actin antibody was purchased from Santa Cruz (Santa Cruz, CA, USA). BET inhibitor I-BET762 (referred to as I-BET), JQ1, and OTX015 and BCL-2 inhibitor ABT-199 were purchased from Selleck Chemicals (Houston, TX, USA). HDAC inhibitor SAHA (vorinostat) was purchased from Sigma-Aldrich (St. Louis, MO, USA).

### Cytogenetic studies by FISH

*MYC*, *BCL2*, and *BCL6* rearrangements were analyzed using break-apart FISH. The MYC (5′ red (R) /3′ green (G)), BCL2 (3′ G/5′ R), and BCL6 (3′ G/5′ R) probes were commercially available from Abbott Molecular (Downers Grove, IL, USA). FISH was performed using standard FISH methodologies [26].

### Assessment of cell proliferation

For thymidine incorporation assay, 1.0 × 10^4^ cells were cultured for 72 h with bromodomain extra-terminal inhibitors (BETi). Before harvesting, cells were pulsed with 1 μCi (0.037 MBq) tritiated thymidine (3H-TdR; Amersham, UK) for 18 h and ^3^H-TdR incorporation levels were determined using a Beckman scintillation counter (GMI). For XTT assay, 0.25 × 10^4^ cells were cultured for 72 h with BETi and XTT was added for 3 h followed by analysis on a SpectraMax plate reader (Molecular Devices, San Jose, CA, USA).

### Cell survival by annexin V/PI staining

5.0 × 10^5^ cells/ml were cultured for 72 h in the absence or presence of BET inhibitors, then stained using 1 μg/ml annexin V–FITC for 30 min at 4 °C. Cells were then washed in annexin V binding buffer and stained with 0.5 μg/ml propidium iodide and analyzed by flow cytometry (FACSCalibur; Becton Dickinson). Data analysis was performed with Flow Jo software (TreeStar).

### Western blotting

Cells were lysed with RIPA buffer for 30 min on ice and lysates cleared by centrifugation, and Western blotting was performed as described earlier [27].

### RNA isolation and RT-PCR

Total RNA was extracted using RNeasy mini kit (QIAGEN, Germantown, MD, USA). cDNA was synthesized using total RNA with SuperScript III First-Strand Synthesis SuperMix (Invitrogen, Grand Island, NY, USA) according to the manufacturer’s instructions. PCR was performed according to the HotStar Taq Master Mix kit instructions. The program consisted of 95 °C for 15 min, 25 cycles of 95 °C for 15 s, 58 °C for 30 s, and 72 °C for 30 s, followed by 72 °C for 10 min. The RT-PCR primers used were as follows:

c-MYC: cMyc-F (5′GGGTAGTGGAAAACCAGCAGCCTC3′)

cMyc-R (5′CATCTTCTTGTTCCTCCTCAGAGTCGC3′).

BCL6: BCL6-F (5′TAACATCGTTAACAGGTCCATGACG3′)

BCL6-R (5′GCCCCGTTCTCACAGCTAGAATC3′)

GAPDH: GAPDH-F (5′GAAGGTCGGAGTCAACGG ATTTG3′)

GAPDH-R (5′ATGGCATGGACTGTGGTCATGAG3′).

### Plasmid constructs and transient transfections

Plasmid DNA (5 μg) for each of the pcDNA3, pcDNA3-cMyc, or pcDNA3-BCL2 (addgene) was transfected using a human B cell Nucleofector kit (Amaxa Biosystems). Briefly, 6 × 10^6^ DLBCL cells were transfected using a U-15 program on a Nucleofector equipment. Two days following transfection, cells were harvested and used for analyses as required.

### Chromatin immunoprecipitation (ChIP)

ChIP assay was performed using the ChIP Assay Kit (EMD Millipore Billerica, MA, USA) with antibodies to BRD4 (Cell Signaling Technology, Cambridge, MA, USA) or IgG following the manufacturer’s instructions. Immunoprecipitated DNA and input were analyzed by PCR using the following primers: c-MYC promoter: F: 5′-AACATGACCAGACTGCCTC-3′and R:5′-CTCAAAGCAAACCTCCTAC-3′; BCL6 promoter: F: 5′-CGTACATTCTCAGCTTATG-3′and R:5′-CTTACGCCTCTCTTTACTG-3′; and BCL2 promoter: F: 5′-CAAGGGGGAAACACCAGAATC-3′ and R:5′-CCCCCAGAGAAAGAAGAGGAG-3′.

### Flow cytometry

Cells (1 × 10^6^ cells) were washed in FACS buffer (PBS containing 2% FBS and 0.05% sodium azide) and incubated with PD-L1-PE conjugated and CD47-FITC conjugated or isotype control antibodies (mouse IgGκ-FITC/PE) (BD Biosciences, San Jose, CA, USA) for 30 min. Cells were washed with FACS buffer and re-suspended in 500 μL FACS buffer, and data were acquired on a FACSCalibur flow cytometer (BD Biosciences). Data was analyzed using FlowJo version10 software.

### Statistics

The data is presented as the mean ± standard error from 3 independent experiments. An unpaired Student *t* test was used for statistical comparisons and a *p value < 0.05 was considered significant.

## Results

### Detection of MYC rearrangements in human BL and DLBCL cell lines

**Fig. 1:**
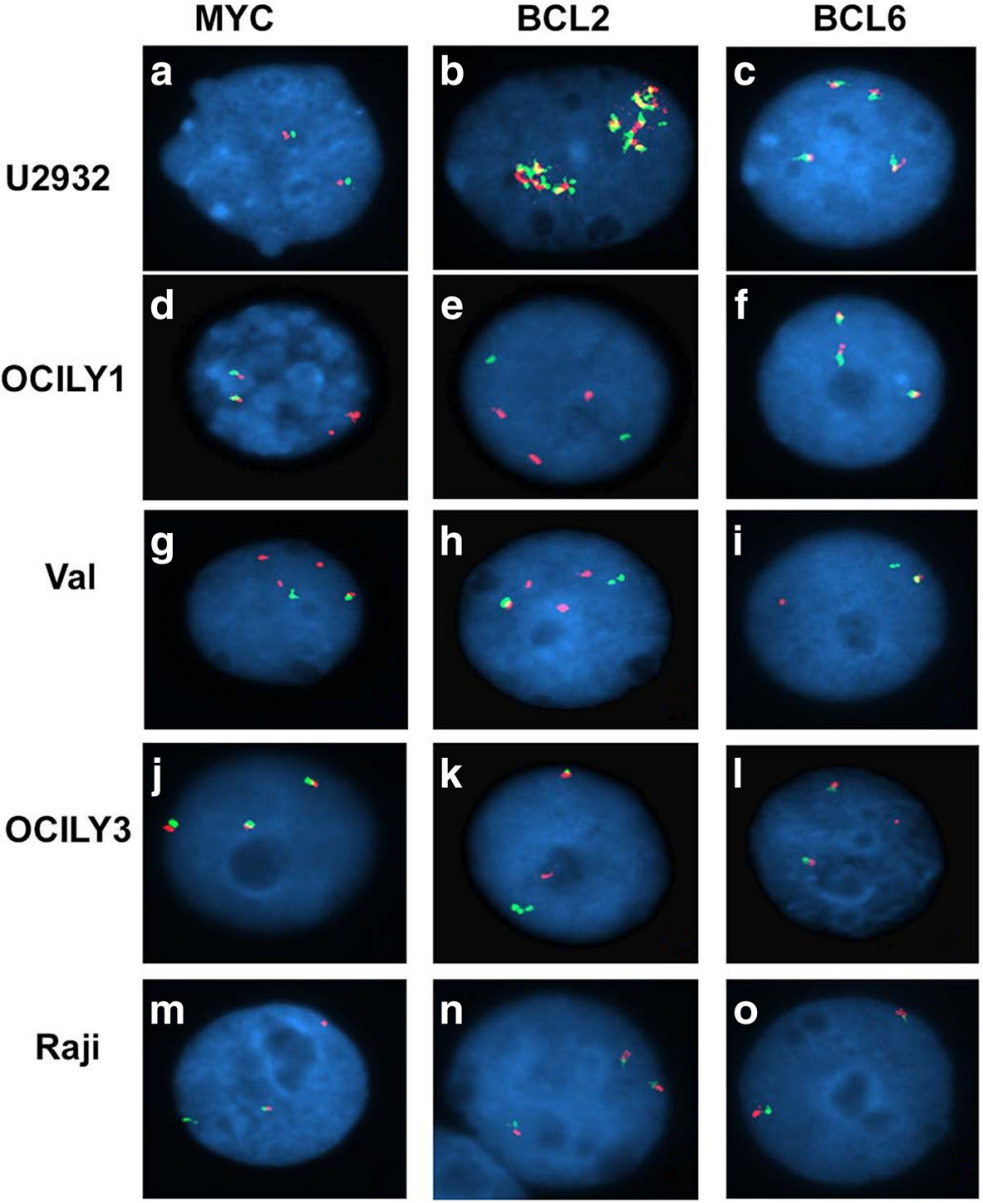
Identifications of double hit and triple hit in DLBCL and BL cell lines by FISH. MYC, BCL2, and BCL-6 rearrangements were detected by FISH using break-apart probes carried out in DLBCL cell lines (*n* = 9) and BL cell line (*n* = 2). Representative images of c-MYC, BCL-2, and BCL-6 FISH in DLBCL and BL cell lines are shown. Images of U2932 (**a**-**c**) indicate a normal 2-fusion (**f**) signal pattern for MYC, amplification of BCL-2, and 4F for BCL6. LY1 MYC 1R1ampR2F, BCL-2 3R2G, and BCL-6 3F (**d**-**f**). VAL MYC 3R1G2F, BCL-2 3R1G1F, and BCL-6 1R1G1F (**g**-**i**). LY3 MYC 3F, BCL-2 1R1G1F, and BCL-6 1R2F (**j**-**l**). Raji MYC 1R1G1F, BCL-2 3F, and BCL-6 2F (**m**-**o**)

We began our studies by evaluating MYC, BCL2, and BCL6 rearrangements in 11 B cell lines by fluorescence in situ hybridization (FISH) using MYC, BCL2, and BCL6 break-apart (BA) probes. Break-apart probes target two areas of a *MYC*, *BCL2*, and *BCL6* gene sequence. Using the BA probe, U2932 showed no MYC rearrangement and showed two normal fusion signals, while translocation positive cells such as Raji, OCILY1, and Val had lost one of the normal fusion signals and had separated red and green signals as shown in Fig. 1. Representative images showing MYC, BCL2, and BCL6 rearrangements are depicted in Fig. 1. Based on the *MYC*, *BCL2*, and *BCL6* rearrangements, DLBCL cell lines were determined to fall into either wild-type MYC (WT-MYC) seen in HBL-1 and U2932 cell lines; single MYC rearrangement with immunoglobulin commonly referred to as single hit (MYC/IG; SH) seen in Raji and Ramos Burkitt lymphoma cell lines; MYC rearrangement with BCL2 gene (MYC/BCL2; DHL) seen in OCILY1, OCILY10, SUDHL2, and DOHH2 cell lines; or MYC rearrangements with both BCL2 and BCL6 genes (MYC/BCL2/BCL6; THL) seen in only VAL cell line (Table 1). We also identified a unique group with no MYC rearrangements and BCL2 and BCL6 translocations (BCL2/BCL6) in OCILy3 and OCILY19 cell lines. These classifications allowed us to examine the effect of BET inhibition in DLBCL cell lines harboring MYC rearrangements occurring with BCL2 and/or BCL6.

**Table 1:** Identification of MYC, BCL-2, and BCL-6 rearrangements in human DLBCL and BL cell lines: c**-***MYC*, *BCL-2*, and *BCL-6* rearrangements were analyzed with break-apart FISH in human DLBCL (*n* = 9) and Burkitt Lymphoma cell lines (*n* = 2)

DLBCL cell lines	cMYC	BCL-2	BCL-6	Status
U2932	Wild-type	Amplification	3–4 copies	WT-MYC
HBL-1	Wild-type	3 copies	Normal	WT-MYC
Raji (BL)	Rearrangement	3 copies	Normal	Single hit
Ramos (BL)	Rearrangement	1 copy	3 copies	Single hit
SUDHL2	Rearrangement/amplification	Rearrangement	3 copies	Double hit
OCILY1	Rearrangement/amplification	Rearrangement	3 copies	Double hit
OCILY10	Rearrangement/amplification	Rearrangement	3 copies	Double hit
DOHH2	Rearrangement/amplification	Rearrangement	Normal	Double hit
VAL	Rearrangement	Rearrangement	Rearrangement	Triple hit
OCILY3	3–4 copies	Rearrangement	Rearrangement	BCL2/BCL6 translocation
OCILY19	3–4 copies	Rearrangement	Rearrangement	BCL2/BCL6 translocation

### Anti-proliferative effect of BET inhibitors in DHL/THL DLBCL cells

MYC overexpression has been shown to be regulated by BRD proteins in multiple cancer types [22, 28]. We examined the sensitivity of BET bromodomain small molecule inhibitors (BETi) such as I-BET-762 (I-BET), JQ1, or OTX015 (OTX) in DHL, THL, and SH cell lines harboring MYC rearrangement with BCL2 and/or BCL6. First, we assessed the effect of low doses (0.5 and 1.0 μM) of the JQ1, I-BET, and OTX on DHL and THL cells along with WT-MYC cells. A limited anti-proliferative effect was seen with the low doses of these inhibitors on these cells and did not reach to LD50 (Additional file 1: Figure S1).

**Fig. 2:**
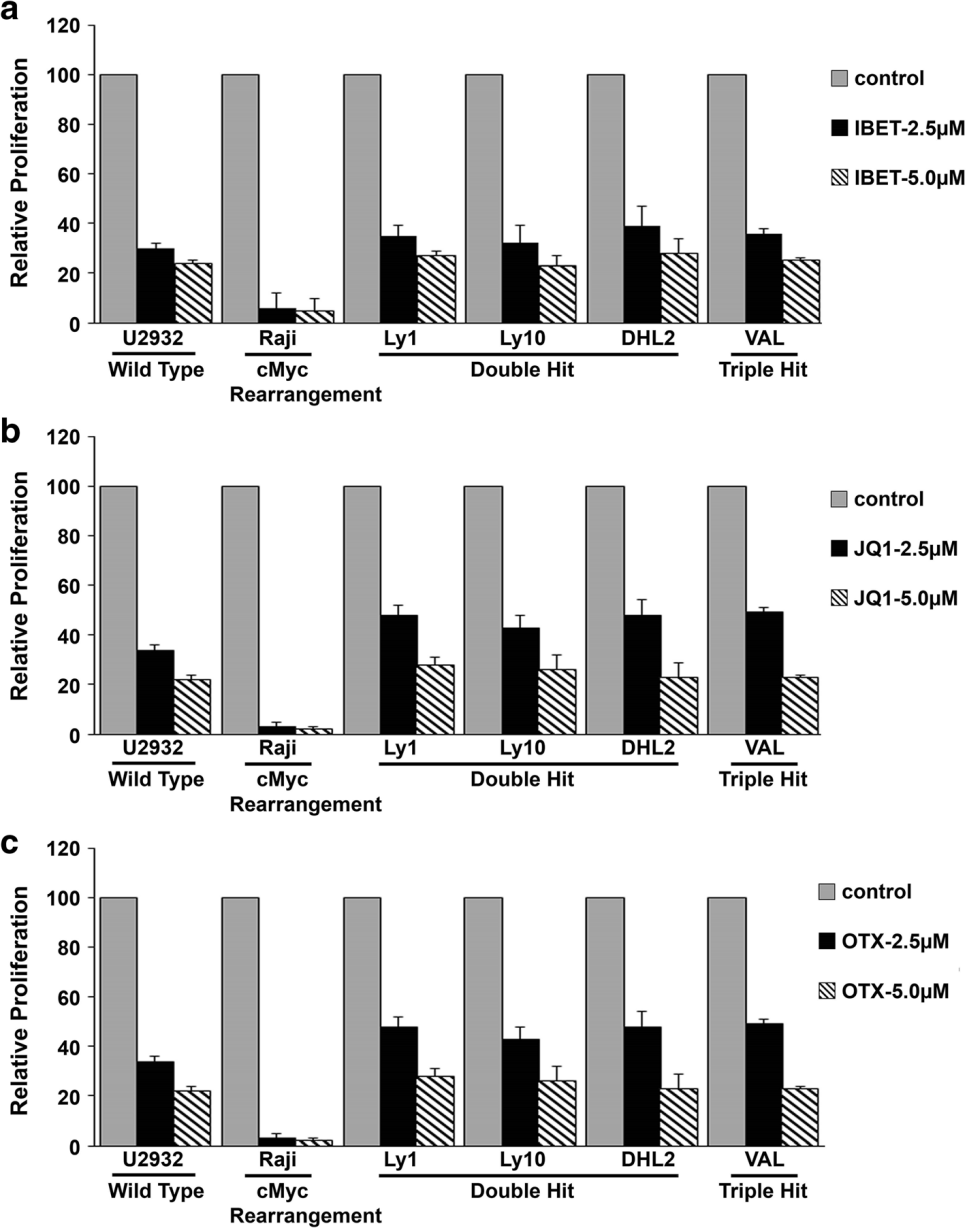
Anti-lymphoma activity of BET bromodomain inhibitors on the DHL/THL cells. **a–c** WT- MYC, SH, DH, and TH harboring DLBCL cell lines were treated with 3 pharmacological BET bromodomain inhibitors I-BET (**a**), JQ1 (**b**), and OTX (**c**) for 72 h and proliferation was assessed by H^3^-thymidine incorporation assay. Data represent mean ± SD from three independent experiments. *p* < 0.05 value was significant for both the concentrations in all the cell lines tested

Next, we increased the doses of JQ1, I-BET, and OTX to 2.5 and 5.0 μM and assessed the anti-proliferative effect in WT-MYC, MYC/IG, BCL2/MYC, MYC/BCL2/BCL6, and BCL2/BCL6 rearrangements. Raji cell line, which harbors a single MYC rearrangement, was the most sensitive to I-BET, JQ1, and OTX with nearly 95% inhibition of thymidine incorporation (Fig. 2a–c). DHL cell lines LY1, LY10, and DHL2 and THL cell line Val were also sensitive to I-BET, JQ1, or OTX, although the overall effect of BETi was less robust than Raji cells but comparable to U2932, which lack MYC rearrangements (Fig. 2a–c). These results suggest that double-hit and triple-hit DLBCL cell lines are sensitive to BET bromodomain inhibitors and the anti-proliferative effect is comparable to cells expressing no MYC rearrangements.

### BET bromodomain regulates MYC and BCL6 but not BCL2 protein in DHL/THL DLBCLs

**Fig. 3:**
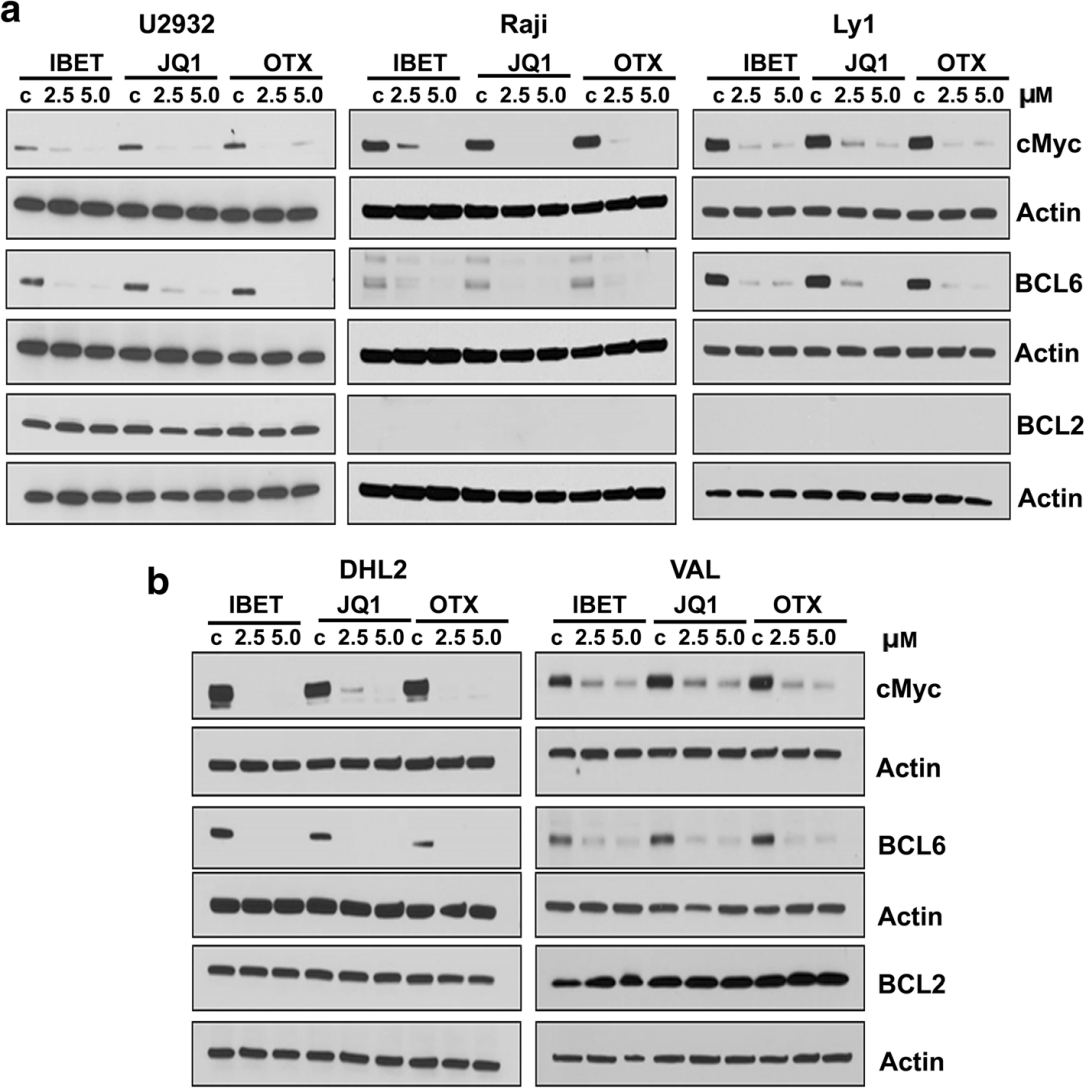
BET inhibition suppresses MYC and BCL6 protein expression in DHL/THL cells. **a**, **b** WT-MYC, SH DHL, and THL harboring DLBCL cell lines were treated with JQ1, I-BET, and OTX, and protein expression was assessed by western blotting. Experiments were repeated three times and a representative western blot image is shown

To gain insight into changes in *MYC*, *BCL-2*, and *BCL-6* expression levels in response to BET inhibitors, WT-MYC (U2932), SH (Raji), DHL (LY1, DHL2), and THL (VAL) expressing human DLBCL lines were treated with various doses of I-BET, JQ1, or OTX. We consistently observed a potent, concentration-dependent decrease in MYC protein expression, across the panel of cell lines tested, suggesting that BETi suppress *MYC* regardless of *MYC* rearrangement status (Fig. 3a, b). Interestingly, unlike MYC expression, the expression of BCL2 was completely insensitive to I-BET, JQ1, and OTX-015 treatment (Fig. 3a, b). However, like MYC expression, BCL6 expression was abolished following treatment with BETi in all the cell lines tested (Fig. 3a, b). These results indicate that MYC and BCL6 (but not BCL2) are regulated by BET bromodomain and can be potentially targeted by BET inhibitors.

### Effect of BET inhibition on *MYC* and *BCL-6* transcription

**Fig. 4:**
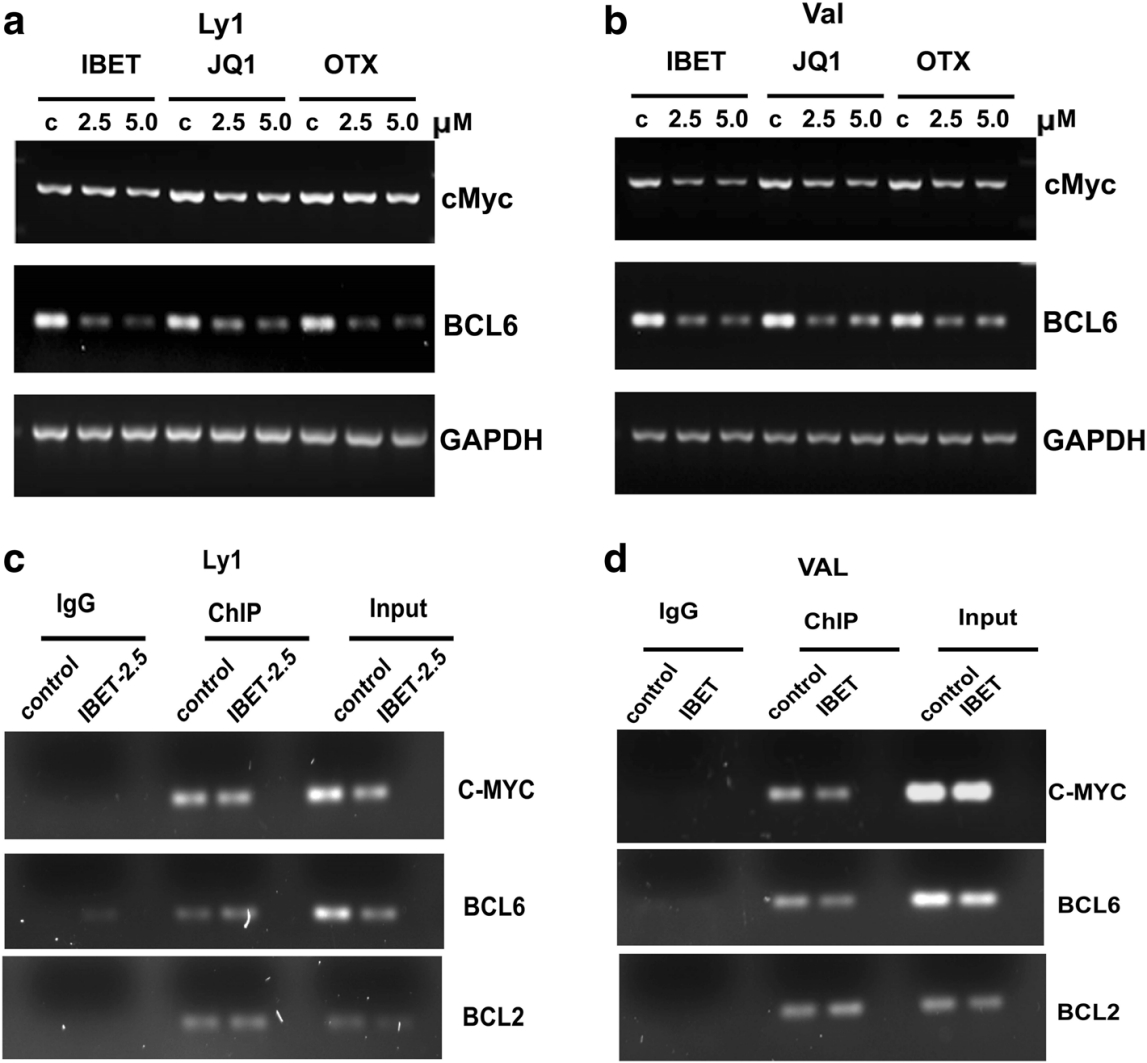
MYC and BCL-6 transcription is regulated by BET bromodomain protein in DHL/THL cells. **a, b** DHL and THL cell lines were treated with BET bromodomain inhibitors I-BET, JQ1, and OTX, and RT-PCR was performed using MYC- and BCL-6-specific primers. **c, d** ChIP assay was performed in the I-BET-treated DHL (LY1) and THL (Val) cells using BRD4 antibody, and RT-PCR was performed using MYC promoter primers. Experiments were repeated three times and a representative image is shown

We next sought to examine the transcriptional changes in *MYC* and *BCL-6 mRNA* induced by BET inhibitors in DLBCL cell lines with MYC rearrangements. Variable effects were observed on *MYC* expression following I-BET, JQ1, and OTX treatment in DHL2 and VAL cell lines. Val cells (THL) showed robust *MYC* mRNA suppression by I-BET, JQ1, and OTX as compared to that observed in LY1 (DHL) (Fig. 4a, b). However, BCL6 mRNA expression was equally suppressed by BETi in LY1 and Val cell lines (Fig. 4a, b). These results suggest that BET protein regulates the expression of both MYC and BCL6 and the impact of BET inhibition on proliferation (Fig. 2) of DHL and THL cells may be manifested by the coordinated loss of MYC and BCL6.

Mechanistically, BETi interfere with *MYC* transcription by physically blocking binding of BRD proteins at regulatory elements that influence *MYC* expression. We analyzed the recruitment of BRD4 to the *MYC* promoter by ChIP assay and found that BRD4 was enriched at the *MYC*, BCL2, and BCL6 promoter regions (Fig. 4c, d). Treatment with I-BET decreased BRD4 binding at the *MYC* promoter in both DHL and THL cell lines. Likewise, BRD4 binding to the *BCL6* promoter region in THL cell line was also decreased. However, BETi had no effect on BRD4 binding to *BCL2* promoter region in any of the cell lines tested (Fig. 4c, d). Taken together, these data indicate that BET inhibition directly modulates *MYC* and *BCL6* (but not *BCL2*) transcription potentially via decreasing BRD4 recruitment to the promoter region of *MYC* and *BCL6*.

### Effect of BET inhibitors on survival of DHL and THL cells

**Fig. 5:**
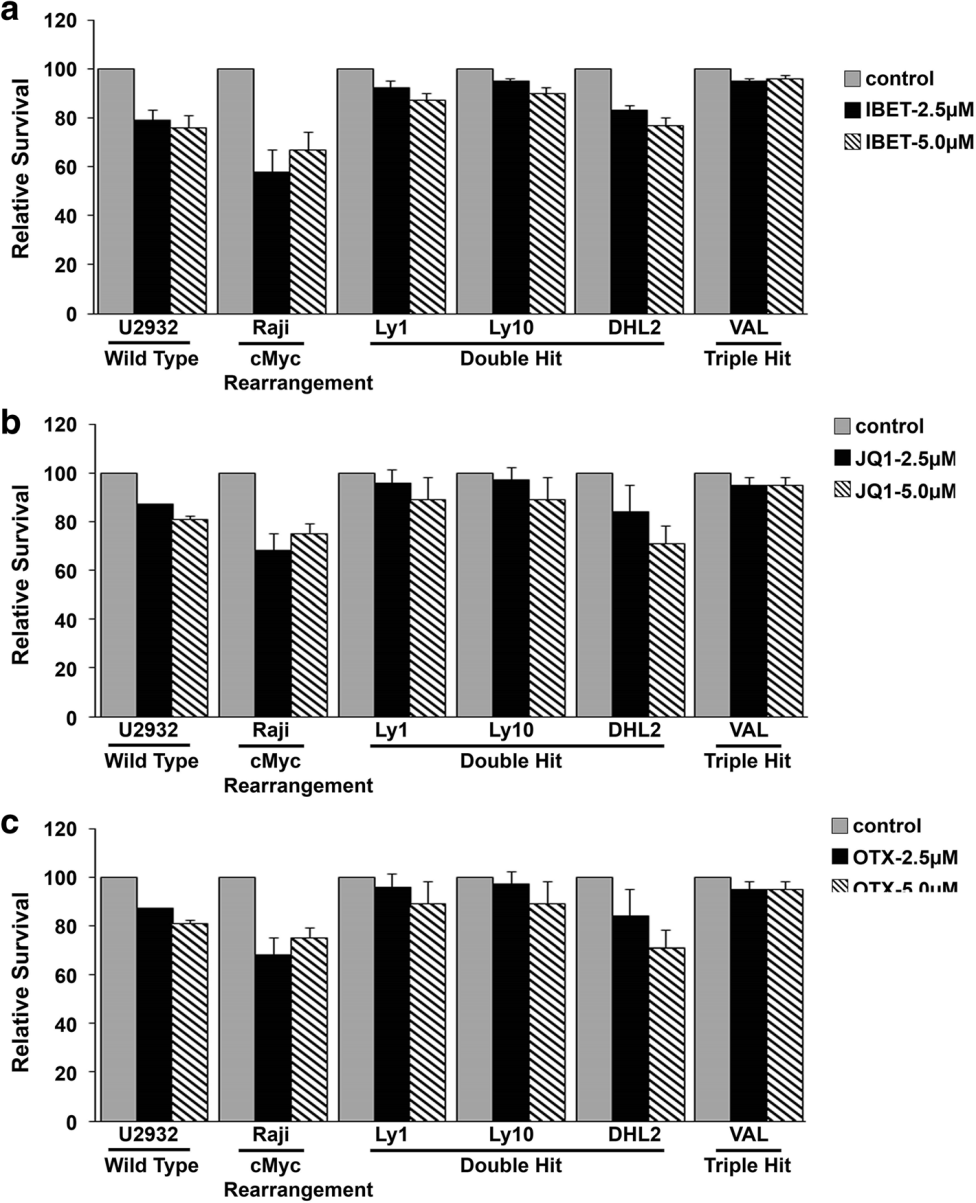
Effect of BET inhibitors on the survival of DHL and THL cells. **a–c** WT-MYC, SH, DHL, THL, and BCL-2/BCL-6 translocation harboring cell lines were treated with 3 different pharmacological BET bromodomain inhibitors I-BET (**a**), JQ1 (**b**), and OTX (**c**) for 72 h and cell survival was assessed by flow cytometry after annexin V/PI staining. Data represent mean ± SD from three independent experiments. *p* < 0.05 value was significant for both the concentrations in all the cell lines tested

We extended the study of BET inhibition to examine the effect on survival of DHL/THL DLBCL cell lines. DLBCL cell lines expressing WT-MYC (U2932), SH (Raji), DHL (LY1, DHL2), and THL (VAL) were treated with varied concentrations of I-BET, JQ1, or OTX015 and then analyzed for survival fraction of cells by excluding annexin V/propidium iodide-stained cells. Surprisingly, unlike cell proliferation data, BET inhibition had only a modest effect on cell survival for cell lines tested (Fig. 5). Cell lines expressing WT-MYC, MYC/IG, or BCL2/BCL6 rearrangements exhibited approximately 20–30% reduction in cell survival at 5 μM dose of BETi, while same dose of BETi in DHL and THL lines demonstrated only 10–15% reduction in cell survival (Fig. 5a–c). There was no significant difference in cytotoxic effects among various BETi used on any given cell line. These results suggest that despite the robust anti-proliferative effects of BETi in DHL/THL cell lines, BETi had only a modest effect on cell viability in DHL and THL DLBCL cells.

### Effect of BET inhibitors on immunoregulatory proteins CD47 and PD-L1 in DHL/THL cells

**Fig. 6:**
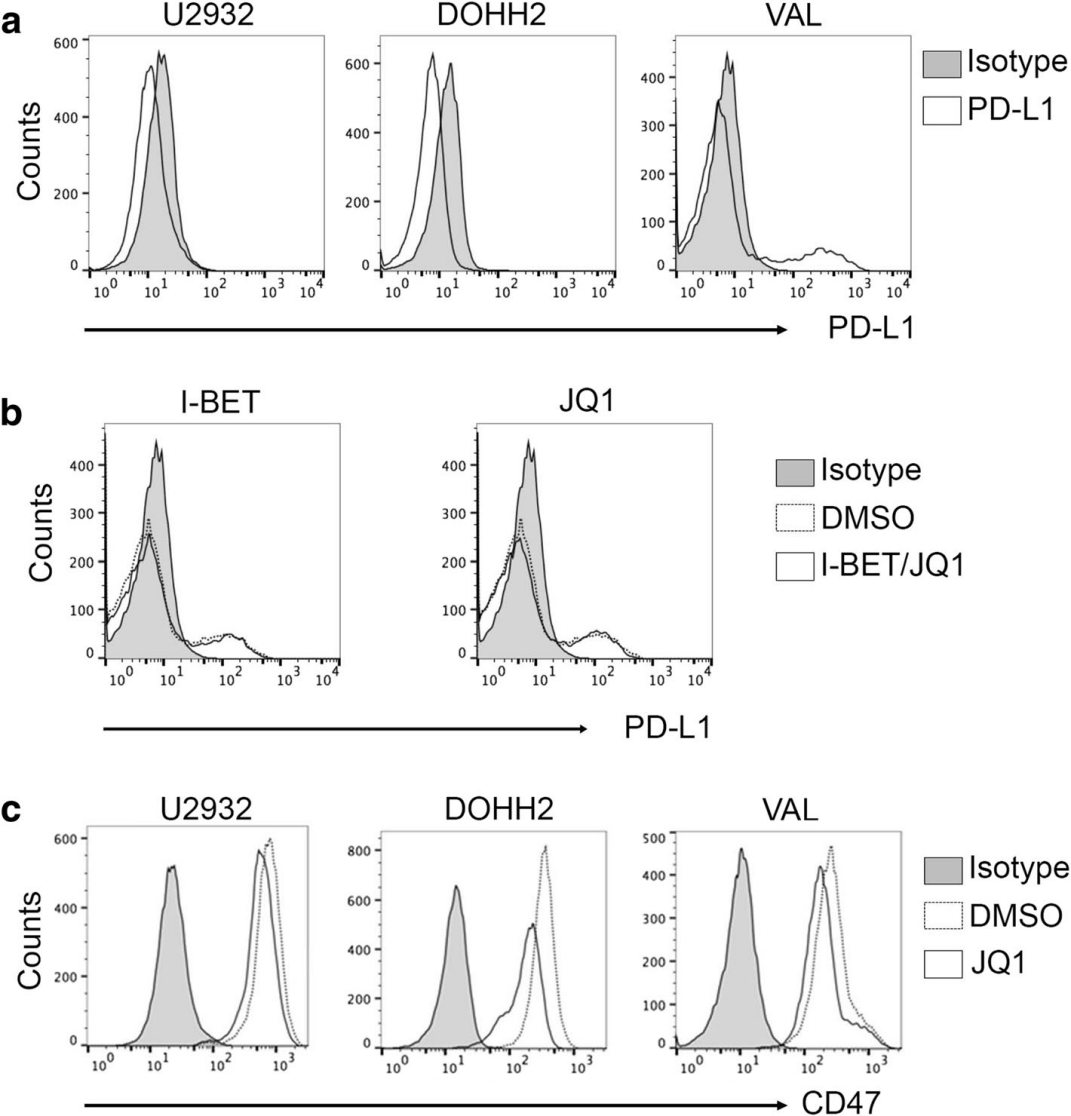
Effect of I-BET and JQ1 on cell surface expression of CD47 and PD-L1 on WT, DHL and THL cells. **a** Cells (0.5 × 10^6^) from wild-type MYC (U2932), double-hit (DOHH2), and triple-hit (Val) B cell lines were harvested, and PD-L1 expression was determined by flow cytometry. **b** Val (0.5 × 10^6^) cells were treated with I-BET (10 μM), JQ1 (10 μM), or DMSO control. After 24 h, cells were harvested and the effects on PD-L1 were investigated by flow cytometry. **c** Cells (0.5 × 10^6^) from U2932, DOHH2, and Val were treated with JQ1 (10 μM) or DMSO control. After 24 h, cells were harvested and CD47 expression was determined by flow cytometry

It has been shown in some cancer cell lines that suppression of MYC with BET inhibitors such as JQ1 reduced programmed cell death ligand 1 (PD-L1) and CD47 expression [29]. We sought to determine the effect of BETi on the PD-L1 and CD47 expression in DHL/THL cells. First, we examined the surface PD-L1 expression in WT-MYC (U9372), DHL (DOHH2), and THL (Val) cell lines by flow cytometry. Surprisingly, WT-MYC and DHL cells did not express surface PD-L1 as compared to isotype control. However, the THL (Val) cells express very low levels of PD-L1 (Fig. 6a). This low level of PD-L1 expression was not changed upon treatment with JQ1 or I-BET (Fig. 6b). When we examined CD47 expression in these cells, we found a robust surface CD47 expression in all cells, which was reduced upon treatment with JQ1 (Fig. 6c). Taken together, these results show that BETi reduces CD47 expression on DLBCL regardless of translocation status while having no effect on PD-L1 expression in THL (Val) cells.

### Effect of co-treatment with inhibitors of BET and HDAC on DHL/THL DLBCL cells

**Fig. 7:**
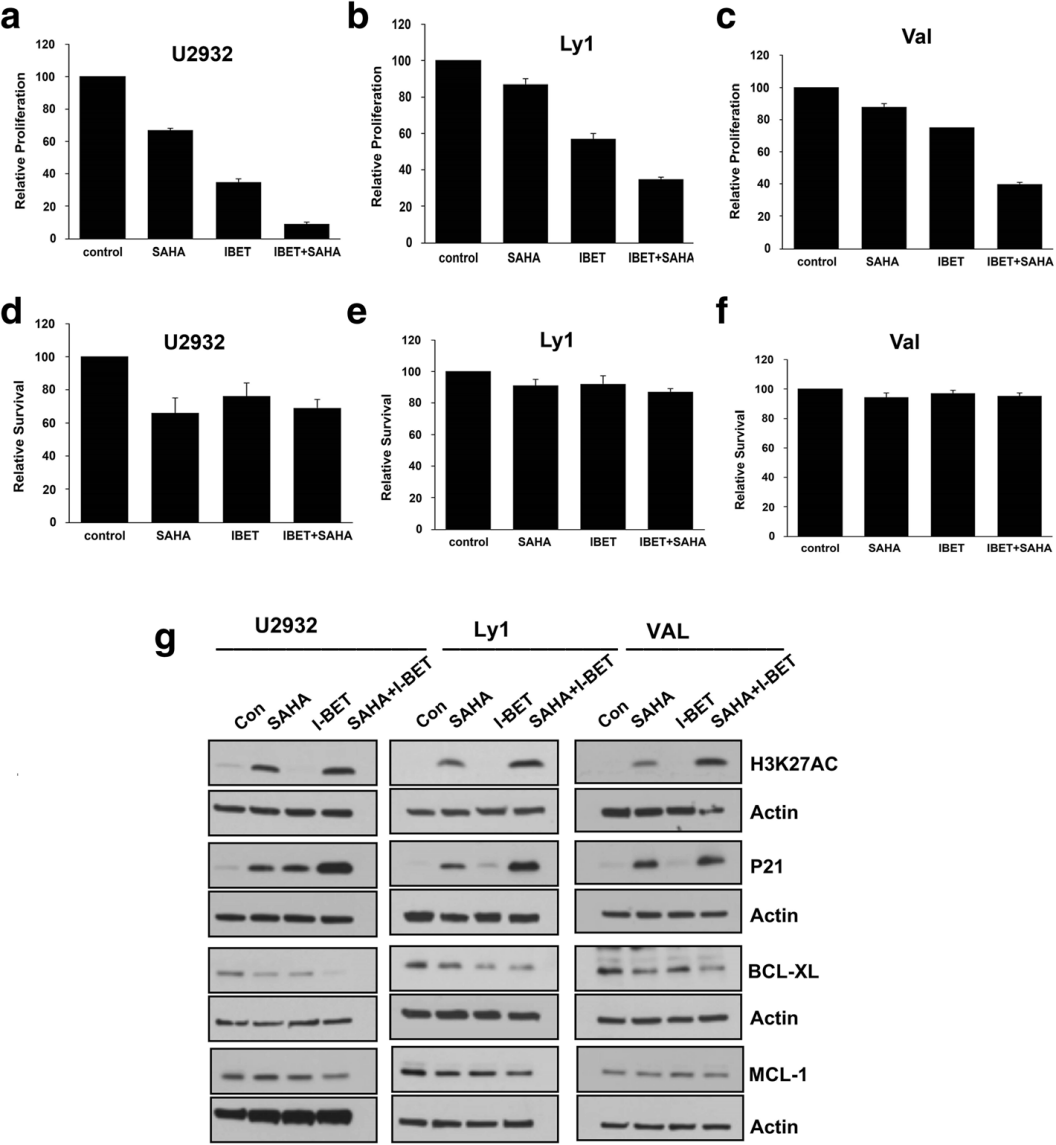
Anti-proliferative effect of combined inhibition of BET and HDAC in DHL and THL. **a–f** WT-MYC (U2932), DHL (LY1), and THL (Val) DLBCL cell lines were treated with I-BET alone, SAHA alone, and combination of both and proliferation and survival analyzed by H^3^-thymidine incorporation and annexin/PI, respectively. **g** The effect on P21, H3K27AC, BCL-XL, and MCL-1 expression was determined by western blotting. Bar represents mean ± SD from three separate experiments. *p* < 0.05, **p < 0.01

BETi mediated transcriptional repression of MYC or BCL6 appears to have little effect on survival of DHL or THL DLBCL cells despite robust anti-proliferative activity. Next, we sought to determine if BETi can sensitize cells to HDAC inhibition. WT-MYC (U2932), MYC/BCL2-(LY1), and MYC/BCL2/BCL6 (VAL) DLBCL cell lines were treated with or without suboptimal concentrations of SAHA, I-BET, or their combination for 72 h and analyzed for cell proliferation and survival. As shown in Fig. 7a–c, the 2.5 μM concentration of SAHA, a Pan-HDAC inhibitor had a significant anti-proliferative effect in U2932 (WT-MYC) cell line; it remained ineffective in LY1 (DHL) or Val (THL), while 2.5 μM I-BET suppressed the proliferation in all three cell lines. Combining SAHA with I-BET further reduced the proliferation in U2932, LY1, and Val with WT-MYC, DHL, and THL status, respectively (Fig. 7a–c). We then evaluated the effect of SAHA and I-BET on cell survival and observed that SAHA or BETi used alone had only a modest decline in cell survival for the WT-MYC cells; the combination had no further change in cell survival in any of the MYC rearrangement DLBCL cell lines tested (Fig. 7d–f).

We next sought to determine the mechanisms of BETi and HDACi combination effect on cell proliferation of DHL and THL cells. We performed the western blot analysis in DLBCL cell lines harboring WT-MYC, MYC/BCL2, or MYC/BCL2/BCL6 rearrangements and tested the impact of combination on histone acetylation (H3K27AC) and the cell cycle regulator P21. As expected, treatment with SAHA increased levels of H3K27 acetylation and P21, combining BETi and SAHA further increased the P21 level in WT-MYC, DHL, and THL cells (Fig. 7g). We have also tested the BETi and HDACi combination effect on antiapoptotic proteins such as BCL-XL and MCL-1. A modest decline in the BCL-XL levels but not in the MCL-1 levels was observed WT-MYC, DHL, and THL cells with either drug alone; however, combination further decreased the BCL-XL level only in WT-MYC cells (Fig. 7g). Interestingly, neither SAHA nor I-BET alone or their combination had any inhibitory effect on BCL2 or BIM protein expression (data not shown). These results suggest that Pan-HDAC inhibitor SAHA synergizes and potentiates anti-proliferative effects of I-BET in DLBCL cell lines via P21 upregulation and histone acetylation despite differences in MYC rearrangement status.

### Effect of combined targeting of BET and BCL2 in DHL/THL DLBCL cells

**Fig. 8:**
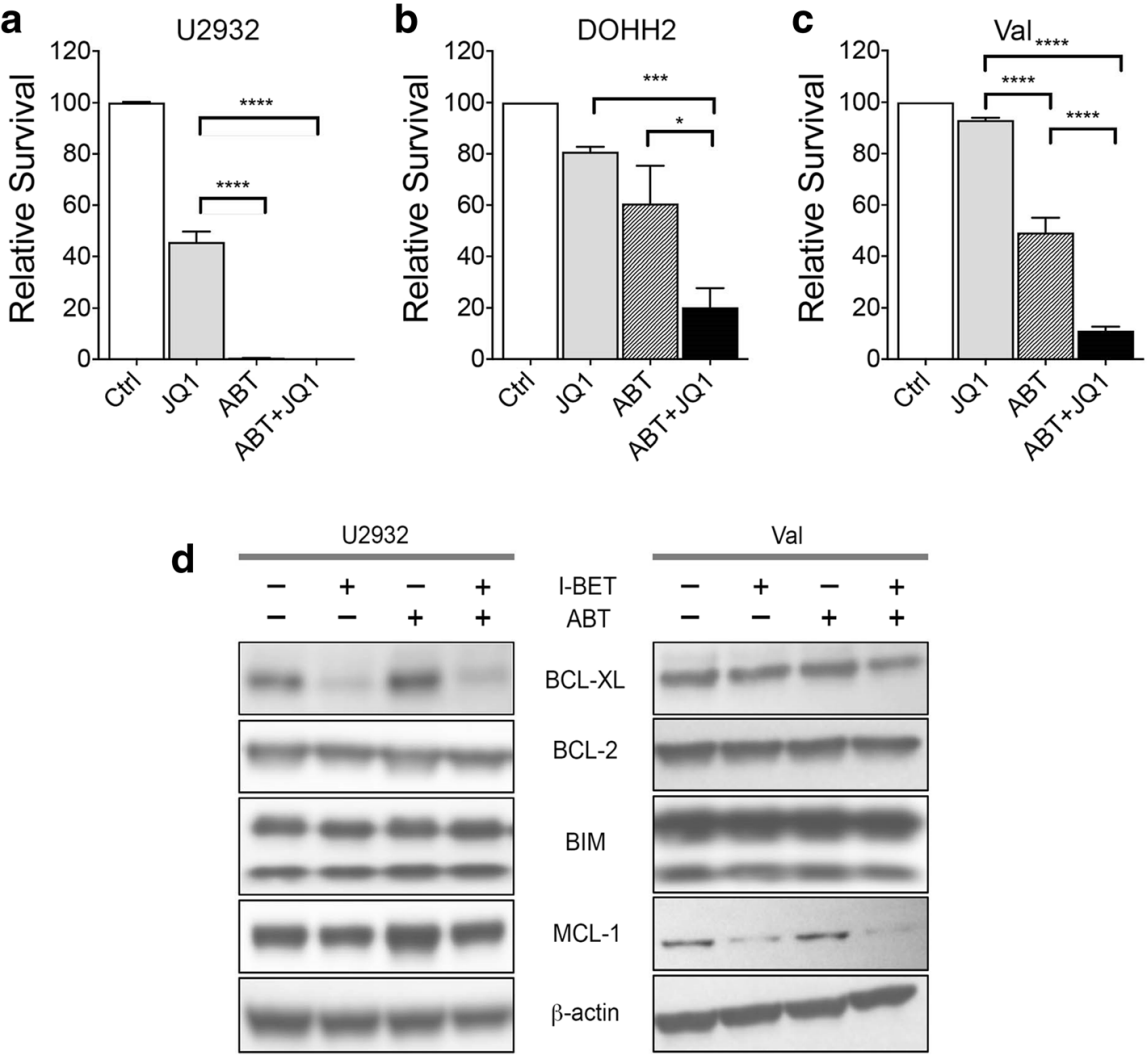
Combinatorial effect of BET and BCL-2 inhibitor ABT-199 on WT-MYC and THL. **a–c** WT-MYC (U2932), DHL (DOHH2), and THL (Val) were treated with JQ1 alone (5 μM), a sublethal dose of ABT-199 alone (500 nM), or a combination of both, and survival assay was performed by annexin/PI staining. **d** WT-MYC (U2932) and THL (Val) cells were treated with I-BET (2.5 μM), ABT-199 (500 nM), and a combination of both, and the effects on BCL-XL, BCL-2, BIM, and MCL-1 proteins were investigated by western blotting using specific antibodies. Bar represents mean ± SD for three replicates from three different experiments. *p < 0.05, **p < 0.01, ***p < 0.005

Several studies have shown the role of *BCL-2* in cancer cell survival and drug resistance [30, 31]. We observed a high basal level of BCL-2 protein in DHL and THL DLBCL cell lines, which was not inhibited by BET bromodomain inhibitors (Fig. 4). Since BCL-2 has been shown to suppress apoptosis in a variety of cell types, we sought to determine if BETi has synthetic lethality with BCL-2 responsible for survival in DLBCL cell lines with various MYC rearrangements. Despite the robust anti-proliferative effect of BETi in DLBCL cell lines, BETi (JQ1) alone had only a limited effect on cell survival, while a pro-apoptotic effect of BCL-2 inhibitor (ABT-199) varied with nearly 90% decreased survival of WT-MYC cell line U2932 and the DHL cell line DOHH2 as compared to a relatively moderate 50% decreased survival in THL cell line Val. Co-treatment with I-BET and ABT-199 had a strong combinatorial effect with nearly complete abolition of cell survival not only in WT-MYC cell line but also DHL (DOHH2) and THL (Val) DLBCL cell lines (Fig. 8a–c). Collectively, these findings demonstrate that treatment with the BETi sensitizes DHL and THL DLBCL cells to BCL-2 antagonist ABT-199.

BCL-2 and related anti-apoptotic proteins BCL-XL or MCL-1 reduce apoptosis by neutralizing pro-apoptotic BCL-2 family members, including BIM and BCL-Xs, and enhance cell survival. To examine the effect of BET and/or BCL2 inhibition on BCL-2 family members, we assessed the effects of suboptimal doses of I-BET and ABT-199 alone or their combination on levels of anti-apoptotic BCL2 family members BCL-XL and MCL-1 and pro-apoptotic member BIM in WT-MYC (U2932) and MYC/BCL-2/BCL-6 (Val) cell lines. The combination of both drugs reduced BCL-XL and MCL-1 levels more than either single agent alone in WT and THL; however, no changes were seen in BIM levels at the doses used in all the cell lines tested (Fig. 8d). Overall, these findings suggest that BETi, in combination with BCL-2 inhibitors, will be effective in targeting DHL or THL DLBCL cells.

## Discussion

The median overall survival of patients with DHL/THL DLBCLs treated with R-CHOP has been reported to be 5 to 24 months [8], and there is an unmet need for alternative therapeutic strategies for this subgroup. MYC protein has been considered “undruggable” and the main approach has been directed at interfering with MYC:MAX dimerization [32, 33]. In various cancers, MYC-dependent transcription requires the critical transcriptional assembly and/or chromatin-modifying enzyme complexes influencing cell division and survival [34, 35]. Bromodomains recognize acetylated lysine in histone tails and activate transcription and function as epigenetic readers. A large number of studies were published showing the efficacy of the BET pharmacological inhibitors, in a B-ALL and multiple myeloma [22, 36]. BET pharmacological inhibitors such as I-BET151 (GSK1210151A) which was reported as a novel BET bromodomain inhibitor that had proved its efficacy in mixed-lineage leukemia (MLL) cell lines at inducing apoptosis and cell cycle arrest [28].

The major challenge for preclinical in vitro studies is the identification of DHL/THL DLBCL cell lines to be used as an in vitro model. We exploited and identified 4 DHLs (OCILY10, OCILY1, DOHH2, and SUDHL2) and 1 THL (Val) along with WT-MYC (U2932 and Karpas 422) DLBCL lines by comprehensive cytogenetic analysis. These DHL/THL cell lines will serve as an in vitro model for preclinical studies. We further our studies by targeting BET protein through several small molecule bromodomain inhibitors including JQ1, OTX015, and I-BET-762 using DHL and THL DLBCL model cell lines. JQ1, OTX, and I-BET at lower concentration (0.05 or 1μM) had a minimum inhibitory effect on proliferation of DHL and THL cells, although at higher concentrations such as 2.5 μM or 5 μM all of the three inhibitors significantly abrogated the proliferation of DHL/THL DLBCL cells and the inhibitory effect was similar to that observed in wild-type MYC overexpressing DLBCL cells. Bhadury et al. have shown the anti-proliferative effect of JQ1 at a much lower concentration in DHL cells from transgenic MYC overexpressing mice [37]. The difference between the concentration might be the proliferation technique or the use of different cell model. Overall, our finding suggests that MYC-expressing lymphoma cells are most probably addicted to the MYC-oncogenic effect and rely on MYC for their growth regardless of MYC rearrangements. Our findings are consistent with the Takimoto-Shimomura et al.; they demonstrated the similar effect of JQ1 on a new DHL cell line [38]. Our finding is consistent with previous studies in hematological disease models of multiple myeloma (MYC-dependent) and acute myeloid leukemia, where it was demonstrated that BET inhibition produces a potent anti-proliferative effect [22, 24, 39]. Additionally, we found that BET inhibition through JQ1 depleted the BRD4 binding at the MYC promoter. This finding is also consistent with previous studies showing a similar mechanism of MYC regulation in multiple myeloma (MM) cells [22]. Our study also demonstrated that CD47 is expressed on the DHL and THL cells and abrogated by JQ1 treatment. This is consistent with the other studies, focusing on the CD47 and PD-L1 as immune targets and regulated by MYC proto-oncogene on MYC-expressing cells [29].

We found that pharmacological inhibition of BET proteins leads to no significant difference in cell survival of DHL, THL cells at the concentration it causes the growth inhibition. The selective anti-proliferative effect of BETi through MYC transcriptional program provides an opportunity to combine BET inhibitors with inhibitors of other signaling pathways. BET inhibitors have been shown to synergize AML cells to HDAC inhibitors [40]. Our results demonstrated here that I-BET synergized with Pan-HDAC inhibitor SAHA and have significant combinatorial effect limited to proliferation but not to survival inhibition in DHL/THL cells. Recently, Badhury et al. also described the synergistic effect of BETi and HDACi in murine and human model of MYC-expressing cells, and our study is somewhat consistent with their study [37]. We also demonstrate that BET inhibition in DHL/THL cells, target BCL6 but has no inhibitory effect on BCL-2 protein. Since BET inhibition had no significant effect on anti-apoptotic protein BCL-2 in DHL and THL cells, we hypothesized that MYC inhibition by BETi could demonstrate synergism with pro-apoptotic agents such as the BCL-2 antagonist ABT-199. ABT-199 is a highly selective inhibitor of BCL-2 that has shown efficacy in CLL [41, 42]. Indeed, combined targeting of BCL-2 using ABT-199 with I-BET showed a synergistic inhibition of DHL/THL cells by inhibiting BCL2 family anti-apoptotic proteins BCL-XL and MCL-1. This finding is consistent with a recent report by Esteve-Arenys et al., who demonstrated that single-agent ABT-199 fails to maintain a significant antitumor activity over time in most *MYC*+/*BCL2*+DHL model; however, this phenomenon was counteracted by the BET bromodomain inhibitor CPI203 [43].

In summary, our findings provide a rationale for using BET bromodomain inhibitors alone or in combination with BCL-2-specific inhibitors as the first-line therapy option for double-hit and triple-hit patients. This study demonstrates that, apparently, there is no distinctive feature of DHL/THL lymphoma with respect to sensitivity to BET inhibitors, and MYC-expressing lymphoma cells are probably addicted to the MYC-oncogenic effect and rely on MYC for their growth regardless of MYC rearrangements. Moreover, our study emphasizes on incorporation of FISH analysis along with MYC expression into the standard diagnostic procedure for prompt and accurate identification of DHL and THL patients to start the BET inhibitor therapy alone or in combination with other therapeutic drugs to improve the outcome.

## Additional file

Additional file 1: **Figure S1.** Effect of low doses of I-BET on the proliferation of DHL/THL DLBCL cells. (A-B) WT-MYC, SH, DHL, THL, and BCL2/BCL6 translocation harboring DLBCL cell lines were treated with low doses of I-BET (0.5 μM and 1.0 μM) and proliferation (A) and survival (B) analyses was performed. (TIFF 1142 kb)

## Data Availability

Not applicable.

## Abbreviations

BETi: Bromodomain extra-terminal inhibitors
DHL/THL: Double/triple-hit lymphomas
DLBCL: Diffuse large B cell lymphoma
GCB: Germinal center B cell
WT: Wild-type
