# Supplementary figures and images for: Targeting MYC activity in double-hit lymphoma with MYC and BCL2 and/or BCL6 rearrangements with epigenetic bromodomain inhibitors
Source: J Hematol Oncol. 2019 Jul 9;12:73. doi: 10.1186/s13045-019-0761-2 (PMC6617630; doi:10.1186/s13045-019-0761-2)

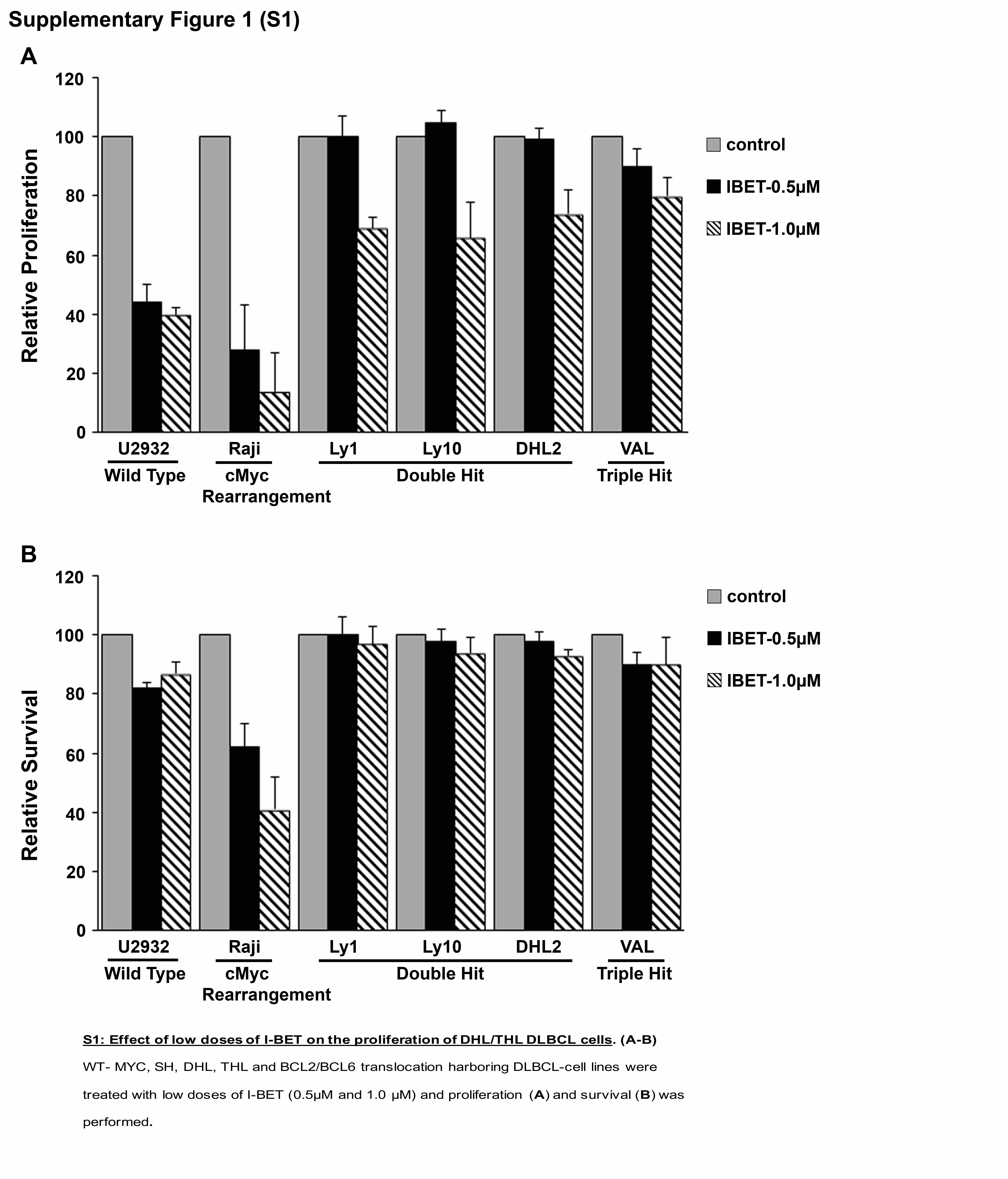

Supplement: Supplementary file 1 — Figure S1. Effect of low doses of I-BET on the proliferation of DHL/THL DLBCL cells. (A-B) WT-MYC, SH, DHL, THL, and BCL2/BCL6 translocation harboring DLBCL cell lines were treated with low doses of I-BET (0.5 μM and 1.0 μM) and proliferation (A) and survival (B) analyses was performed. (TIFF 1142 kb) [file 13045_2019_761_MOESM1_ESM.tiff]
